# “A Friend Among Strangers” or the Ambiguous Roles of Runx2

**DOI:** 10.3390/biom14111392

**Published:** 2024-10-31

**Authors:** Kseniia Azarkina, Ekaterina Gromova, Anna Malashicheva

**Affiliations:** Laboratory of Regenerative Biomedicine, Institute of Cytology, Russian Academy of Sciences, 194064 Saint-Petersburg, Russia

**Keywords:** Runx2, osteogenic differentiation, notch signaling pathway, calcification, tissue specificity of Runx2

## Abstract

The transcription factor Runx2 plays a crucial role in regulating osteogenic differentiation and skeletal development. This factor not only controls the expression of genes involved in bone formation, but also interacts with signaling pathways such as the Notch pathway, which are essential for body development. However, studies have produced conflicting results regarding the relationship between Runx2 and the Notch pathway. Some studies suggest a synergistic interaction between these molecules, while others suggest an inhibitory one, for example, the interplay between Notch signaling, Runx2, and vitamin D3 in osteogenic differentiation and bone remodeling. The findings suggest a complex relationship between Notch signaling and osteogenic differentiation, with ongoing research needed to clarify the mechanisms involved and resolve existing contradictions regarding role of Notch in this process. Additionally, there is increasing evidence of contradictory roles for Runx2 in various tissues and organs, both under normal conditions and in pathological states. This diversity of roles makes Runx2 a potential therapeutic target, offering new directions for research. In this review, we have discussed the mechanisms of osteogenic differentiation and the important role of Runx2 in this process. We have also examined its relationship with different signaling pathways. However, there are still many uncertainties and inconsistencies in our current understanding of these interactions. Additionally, given that Runx2 is also involved in numerous other events in various tissues, we have tried to comprehensively examine its functions outside the skeletal system.

## 1. Introduction

The transcription factors of the RUNX family are characterized by their conservative Runt domain, first studied in the *runt* gene in *Drosophila melanogaster*, which plays an important role in development during embryogenesis [1,2]. Subsequently, the elucidation of the functions of a member of this Runx2 family in vivo by different research groups led to the conclusion that Runx2 plays a crucial role in osteoblast differentiation and bone formation [3,4]. According to these studies, mice with a homozygous mutation in *Runx2* died immediately after birth, while they completely lacked bone formation, but cartilage developed almost normally. Mice with a heterozygous mutation in the *Runx2* gene developed clavicular cranial dysplasia, which is characterized by defects in the skull bones, complete or partial absence of clavicles, as well as additional teeth and low growth [4,5,6].

Osteogenic differentiation occurs during bone formation with direct (intramembranous) ossification, when mesenchymal cells differentiate and form bone tissue, or indirect (endochondral) ossification, when cartilage is replaced by bone tissue [7]. There are also separate processes of bone remodeling, regeneration, and growth that take place throughout a person’s life. Osteogenesis is primarily characterized by calcification of the extracellular matrix (ECM), which normally occurs in human bones, teeth, and cartilage and serves to strengthen these tissues [8]. However, calcification can also occur in other tissues—aging or certain diseases and injuries can lead to the formation of calcium deposits in soft tissues and organs, as well as in the cardiovascular system [9].

During intramembranous ossification, Runx2 regulates the differentiation of mesenchymal cells into osteoblasts, which produce ECM. This ECM then mineralizes, forming an ossification center. The proliferation of the ossification center around capillaries and other cells leads to the formation of red bone marrow, while osteoblasts in the surface layer become the periosteum [10]. In contrast, during endochondral ossification, the transcription factor Runx2 triggers the maturation of cartilage chondrocytes and promotes their proliferation by inducing Indian hedgehog (Ihh); then, Runx2 induces transdifferentiation of terminal hypertrophied chondrocytes into osteoblast progenitors [11].

During remodeling, the old bone is resorbed and a new one is formed [12]. The main types of cells controlling bone remodeling are osteoclasts, osteoblasts, and osteocytes [9]. Osteoclasts, multinucleated macrophages that are responsible for the resorption of damaged or old bone, acidify the problem area of the bone, thereby effectively dissolving minerals, and then remove degraded components from the cell [13,14]. To form a new bone, calcium and orthophosphate-containing compounds are isolated from the bloodstream by *osteoblasts*, cells of mesenchymal origin, which, in turn, deposit a structural ECM called an osteoid, consisting mainly of type I-oriented collagen fibers. This osteoid is then mineralized. Some non-collagen proteins and other small molecules are also secreted into the osteoid to regulate the mineralization process [15]. Many osteoblasts further differentiate into osteocytes, which are cells with dendritic processes located within the mineralized osteoid. Runx2 and other transcription factors control osteoblast differentiation [16,17,18].

Next, we consider the mechanisms of osteogenic differentiation, primarily the effect of Runx2 on its molecular targets at different stages of osteogenic differentiation, as well as the relationship of this factor with the main interacting signaling pathways: Wnt, TGFβ/BMP, FGF, vitamin D3, and Notch.

However, Runx2 has not only been observed in osteogenesis—it plays an important role in other processes (both normal and pathological) in various organs and tissues. A separate chapter of this review is devoted to the tissue specificity of Runx2.

## 2. Mechanisms of Osteogenic Differentiation

Osteoblasts originate from mesenchymal stem cells (MSCs) [19]. Mesenchymal progenitors are able to differentiate in the osteo-, chondro-, and adipogenic directions, or into myoblasts. Interestingly, mature osteoblasts retain some surface markers of MSCs, including CD90 and the intercellular adhesion molecule ICAM-1 [20].

Osteoblasts have a pronounced ability to synthesize ECM. The commitment of mesenchymal progenitors and preosteoblasts is regulated by the transcription factor Runx2, which is considered the main regulator of osteogenic osteoblast differentiation and bone formation. This regulation is also influenced by its target gene *BGLAP*, which encodes the protein osteocalcin (OCN). OCN interacts with ECM and leads to its mineralization [21]. Differentiation occurs in three stages, and each stage is determined by a distinct set of molecular markers [22].

During osteogenic differentiation of rat bone marrow MSCs, the level of the Runx2 transcription factor decreases, while the Osterix transcription factor accumulates in cells. The peak of Osterix expression was recorded on the 14th day of cell culture, which indicates that it is a late marker of osteogenic cell transition [23]. Osteogenesis in vitro is initiated by several factors, for example, using dexamethasone, which triggers the Wnt/β-catenin-dependent expression of Runx2 [24], Osterix, and other proteins of the extracellular matrix of bones [25]. And the synthesis of Col1 is controlled by ascorbic acid and β-glycerophosphate [26]. In addition to inducing osteogenic differentiation, Runx2 also inhibits the differentiation of MSCs towards the adipogenic lineage [27]. BMP2 can also induce Osterix expression independently of Runx2 [28].

After the commitment process, MSCs differentiate into preosteoblasts. A preosteoblast has the shape of an ellipse with an elongated nucleus and is capable of proliferation. They express Runx2, D1x5, MSX2, P2Y4, and P2Y14 [29,30], as well as several osteoblast markers such as ALP, Col1, and OPN, and their expression increases as cells mature.

The regulation of osteoblasts and the control of their maturation are provided, among other things, by microRNA molecules. MicroRNA production systematically increases from the moment of induction of osteogenic differentiation until the 6th day of cultivation. During this period, a dynamic increase in the level of microRNAs can be observed, which continues until the 12th day of osteogenic cell differentiation [31]. In addition, microRNAs have been shown to activate the expression of pro-osteogenic markers such as *Runx2* and *Osterix*, as well as the gene responsible for producing ALP. Therefore, microRNAs have osteoinductive and regulatory effects while inhibiting adipogenic differentiation in mouse and human cells [31].

Mesenchymal progenitors can differentiate into immature osteoblasts, which are controlled by Runx2 and Osterix, as well as β-catenin. β-catenin enhances the expression of Runx2 [32]. Osterix, containing a zinc finger domain, is an osteoblast-specific transcription factor and triggers the expression of genes involved in mineralization in both immature and mature osteoblasts. It also controls the balance of chondrocytes and osteoblasts in the tissue and inhibits the differentiation of chondrocytes [33].

Immature osteoblasts produce certain proteins that prevent their differentiation into chondrocytic and adipocytic lines, including alkaline phosphatase (ALP), type I collagen (Col1), bone sialoprotein (BSP), and osteopontin (OPN, SPP1), and genes encoding these proteins are targets for Runx2 binding [32,34].

ALP is an early marker of differentiation, which plays an important role in calcification [34]. Col1 mediates cell adhesion, proliferation, and differentiation of the osteoblast phenotype and, therefore, can be considered as an early marker of osteodifferentiation [35]. Bone sialoprotein is a non-collagenous protein that plays a role in the process of calcium deposition into the ECM. It could also be used as a marker of osteoblast differentiation [36]. OPN is an adhesive glycophosphoprotein that is released in bones by osteoblasts and osteoclasts [35]. Together with BSP, OPN is necessary not only for the mineralization process, but also for the regeneration of mineralized tissue [37,38]. Additionally, OPN has been shown to inhibit vascular calcification [39].

OCN is the most abundant non-collagenous protein found in bones and can serve as a suitable marker of osteogenic maturation [40]. It is considered a later indicator of osteogenic differentiation and an endpoint in the regeneration of hard tissues [35]. When mature osteoblasts are finally embedded in the bone matrix and become osteocytes, they express high levels of OCN [32]. In order to continue bone maturation, Runx2 expression is suppressed, as it inhibits the maturation of osteoblasts and maintains them in an immature state [41]. Consequently, ALP expression decreases, while OCN expression increases [42].

Osteocytes release fibroblast growth factors (FGFs), bone morphogenetic proteins (BMPs), receptor activator of nuclear factor kappa-B ligand (RANKL), osteoprotegerin (OPG), and sclerostin (SOST). The most common paracrine factors causing the induction of osteogenic cell differentiation are BMP-1, FGF, insulin-like growth factor (IGF), and stromal cell-derived factor 1 (SDF-1) [43,44]. The activity of osteoblasts is regulated by FGF and BMP, and osteoclast differentiation is induced by RANKL [45]. OPG inhibits osteoclastogenesis and protects the skeleton from excessive bone resorption by binding to RANKL and thus preventing activation of the RANK receptor [46]. There is evidence that the OPG–RANK–RANKL system controls the migration of cancer cells, thus contributing to the development of bone metastases [47]. Thus, osteocytes control bone resorption and deposition, affecting the activity of osteoclasts and osteoblasts [45].

All of the above regulatory features of osteogenic differentiation are inherent in cells in a normal state. In addition, there are processes of age-related and pathological calcification, during which changes/violations of the mechanisms of normal regulation occur, in which the transcription factor Runx2 plays the same key role (see the Section 4 of this review).

Thus, Runx2 is a regulator of osteogenic cell differentiation in normal conditions during the development and maintenance of tissue homeostasis, as well as pathological tissue changes, for example, during vascular calcification [48].

## 3. Runx2

The Runx2 protein belongs to the RUNX family of transcription factors, which includes three members: Runx1, Runx2, and Runx3. All of them contain a Runt DNA-binding domain that has a length of 128 amino acids [49]. This domain is responsible for DNA binding and heterodimerization with the non-DNA binding β-subunit (CBFβ)—this complex is more stable and enhances the activity of Runx, thereby stimulating the activity of osteoblasts (in the case of binding to Runx2) [1,49,50].

The expression of the *Runx2* gene is regulated by two promoters, and this leads to the formation of two different mRNA isoforms [41]. Type I and type II isoforms of Runx2 have similar functions, although they differ in their dependency on the CBFβ cofactor [51]. Initially, it was believed that the type I isoform was specific to T-cells [49], while the type II isoform was considered specific to osteoblasts [6,21,52]. However, it has been shown that both types of Runx2 protein are expressed in normal osteoblasts and terminal hypertrophied chondrocytes, and only the type I isoform is expressed in undifferentiated mesenchymal stem cells, preosteoblasts, and chondrocyte progenitors [53].

Both isoforms also exist in lung tissue, while the type I isoform can be found in the heart, brain, spleen, and skeletal muscles [54]. In addition, there is evidence of a type III isoform of Runx2 mRNA in mice and rats. It has the same reading frame as the type II isoform, but the initiation of translation occurs at different sites [21,55,56]. All three isoforms of Runx2 participate in the stimulating effect of osteoblast differentiation, although their specific functions are not fully understood [55]. The type II mRNA isoform inhibits osteoblast maturation, affecting the synthetic abilities of cells, and also reduces the differentiation potential of osteoblasts in mice. The type I isoform has a weaker inhibitory effect on osteoblast maturation and further transition to osteocytes [51].

The study of heterozygous and homozygous models of Runx2 mutant mice showed that Runx2 also plays a significant role in the expression of osteoblast-specific genes [21]. For example, Runx2 triggers the expression of osteocalcin (*BGLAP*) by binding to the OSE2 element of the *BGLAP* promoter region. Other marker genes of osteoblasts, such as *ALP*, *Col1*, *BSP,* and *OPN*, also have OSE2-like elements that are similarly regulated by Runx2 [41,57,58].

The data obtained after CHIP sequencing demonstrated that the Runx2 factor binds to the distal region of the enhancer and the proximal region of the promoter of the target gene [59,60]. Using the example of *SPP1*, the Chip-seq data demonstrate a classic target of the Runx2 transcription factor, since it has several binding sites near the *SPP1* promoter [61]. In addition, several additional Runx2 protein binding sites were identified at the SPP1 locus. This confirms the idea that Runx2 targets genes are potentially regulated by multiple sites, most often located distally from the promoters of these genes. Runx2 also regulates the distal enhancer of the SP7 gene, which encodes Osterix. Chromatin with osteoblast-specific affinity was also detected [62].

It is known that the activity and expression of the *Runx2* gene could be regulated by a number of hormones and cytokines. For example, an increased dose of glucocorticoids leads to osteoporosis in mice and humans, and, in particular, to inhibition of Runx2 [63]. And proinflammatory cytokines, although they do not directly affect Runx2, have a negative effect on osteoblast differentiation and lead to increased osteoclastogenesis [64]. It is also known that Runx2 is capable of autoregulation of its expression [41]. In addition, several complex pathways have been discovered that affect the activity and expression of the *Runx2* gene, which we will consider further.

### 3.1. Wnt/LRP5/β-Catenin Signalling Pathway and Runx2

The Wnt/LRP5/β-catenin signaling pathway is crucial for osteoblast differentiation and bone mass maintenance [65,66,67,68]. Wnt ligands, binding to the Frizzled receptor and LRP5/6 coreceptors, stabilize cytosolic β-catenin, preventing its phosphorylation—nonphosphorylated β-catenin moves to the nucleus and regulates the expression of target genes, thereby stimulating osteoblast proliferation [69]. Mutations in *LRP5* can lead to an increase or decrease in bone mineral density [70]. Studies show that β-catenin and Lef1 (the nuclear effector of this signaling pathway) synergistically modulate the activity of *Runx2* [71]. Moreover, SFRP1, acting as a soluble modulator of Wnt signals, can disrupt osteoblast functions due to the destabilization of β-catenin. Mice without SFRP1 demonstrate enhanced Wnt signaling, as well as a high bone mass phenotype characterized by 2-5-fold activation of *Runx2* promoter activity, 7-8-fold induction of endogenous Runx2 mRNA, and a significant increase in OCN levels. All of the above indicates that *Runx2* is a target of canonical Wnt signaling during bone formation [72,73]. In general, in vitro and in vivo studies have shown that blocking the secretion of Wnt pathway ligands in mature osteoblasts disrupts the processes of osteogenic differentiation and bone formation [74].

### 3.2. TGFβ/BMP/Smads Signaling Pathway and Runx2

Opposite effects on osteogenesis are attributed to the TGFβ and BMP factors. Bone morphogenetic proteins (BMP) are necessary for the rapid formation of the skeleton in newborn animals. Overexpression of BMP antagonists by osteoblasts of postnatal mice leads to osteopenia, bone fragility, and spontaneous fractures [75,76]. Recombinant human BMP2 induces osteoblast phenotype in both the non-osteogenic mouse pluripotent cell line C3H10T1/2 and C2C12 mesenchymal progenitor cells [77,78], and also stimulates osteoblast maturation of the rat osteoblast progenitor cell line ROB-C26 [79]. TGFβ, in turn, can inhibit osteoblast differentiation depending on its stage: TGFβ stimulates proliferation and early differentiation of osteoblasts, but inhibits terminal differentiation [80].

The mechanism of the BMP2 factor is described as follows: in vitro studies show that BMP2 binds to BMP receptors to phosphorylate proteins Smad 1, 5, and 8, then forms a complex with Smad 4. This complex binds to target genes and modifies their expression to stimulate multipotent cells to differentiate into osteoblasts [81,82,83]. A similar signaling mechanism operates in the case of TGF-β, but instead of phosphorylation of the Smad 1, 5, and 8 proteins after they bind to receptors, the Smad 2 and Smad 3 proteins become activated.

BMP does not seem to induce Runx2 expression in MSCs, although it is often assumed that Smad proteins directly regulate Runx2 expression; however, the mechanism of their interaction is not entirely clear. It is assumed that BMP signaling promotes *Dlx5* expression in osteoblasts, and Dlx5 then induces *Runx2* expression in osteoprogenitors. Further, Runx2 and the Smad complex can physically interact in MSCs, jointly regulating the transcription of target genes, and thus, it is possible that Runx2 affects the implementation of BMP functions, and not vice versa [84].

### 3.3. FGFs and Runx2

Another family of growth factors is known as a positive regulator of Runx2 expression—fibroblast growth factors (FGFs) [85]. Accelerated bone formation and osteoblast proliferation were observed in mice carrying the Pro250Arg activating mutation at the Fgf 1 receptor (Fgfr1). In addition, this mutation led to increased expression of *Runx2* and other osteodifferentiation genes compared to wild-type mice. It was also shown that in vitro treatment of C3H10T1/2 cells with FGF2 and FGF8 ligands induced *Runx2* expression. On the other hand, Runx2 is able to induce the expression of *Fgfr1*, *Fgfr2,* and *Fgfr3* through direct regulation of their promoters [86].

### 3.4. 1,25-(OH)_2_-Vitamin D3 Signaling Pathway and Runx2

1,25-(OH)2-vitamin D3 (VD3) induces the expression of *Runx2* in human bone cells [87] and enhances the activation of the *BGLAP* gene in rat osteoblasts through its nuclear receptor, VDR. The *BGLAP* gene promoter contains three Runx2 recognition sites—A, B, and C. Sites A and B are located on the sides of the vitamin D-responsive element (VDRE). Mutations of these sites lead to the elimination of VD3-dependent enhancement of osteocalcin transcription, which indicates a functional relationship between VDR and Runx2 factors [88].

However, in studies on MC3T3-E1 and ROS 17/2.8 cell lines that expressed *VDR*, the activity of the *Runx2* promoter was significantly reduced by VD3, and this effect was eliminated by mutation of the VDRE site in the *Runx2* promoter. Similarly, forced expression of VDR in ROS 24.1 cells led to a sharp decrease in the activity of the *Runx2* promoter [89]. In vitro studies also showed that deletion in the *VDR* gene enhanced osteoblast differentiation, including increased alkaline phosphatase activity, mineralized matrix formation, BSP expression, and an increase in the number of colony-forming units of osteoblasts (CFU-OB). These data may indicate that VDR plays a negative role in the differentiation of osteoblasts in vitro [90].

More recent work on the study of the effects of VD3 and VDR has demonstrated that VD3 can be both a stimulating and an inhibitory agent in osteogenesis processes, and this depends on the level of VDR and the availability of VD3 to cells [91]. In this study, the effects of physiological and pharmacological doses of VD3 on cranial osteoblasts of mice with three different genetic features characterized by normal VDR levels, overexpression of VDR, and knockout of VDR were compared. The researchers concluded that the early stages of osteoblast differentiation and VCM mineralization do not depend on the VD3/VDR system; however, VDR may be especially important in the late stages of differentiation—cells with VDR knockout showed a low level of expression of markers of mature osteoblasts/osteocytes during long-term cultivation under conditions of osteogenic differentiation, whereas cells with overexpression of VDR had more mature phenotypes. At the same time, the pharmacological dose of VD3 reduced the level of VCM mineralization in cells with normal and elevated VDR levels, while it did not change that in cells with knockout. However, under different conditions, the relative level of Runx2 mRNA did not change, although the mRNA level of some osteogenic genes, for example, osteocalcin, differed significantly.

The connection between Runx2 and the above-mentioned signaling pathways during osteogenic differentiation has been extensively studied [11,69,84,92]; however, there are still some gaps in our understanding of the mechanisms underlying their interaction. Questions remain regarding the impact of these pathways on Runx2 in proliferating cells, and it is unclear whether BMP regulates Runx2 or vice versa. It is also puzzling to have opposite data on the effects of vitamin D3 and its receptor on cell differentiation. Meanwhile, there is another significant signaling pathway known to be associated with Runx2. However, the exact mechanisms by which this pathway affects Runx2 function and osteogenic processes remain unclear.

### 3.5. Notch Signaling Pathway and Its Effect on Osteogenic Differentiation

There is conflicting evidence regarding the role of the Notch signaling pathway in stimulating or inhibiting osteogenic differentiation and, therefore, the association between Notch and Runx2.

**Figure 1:**
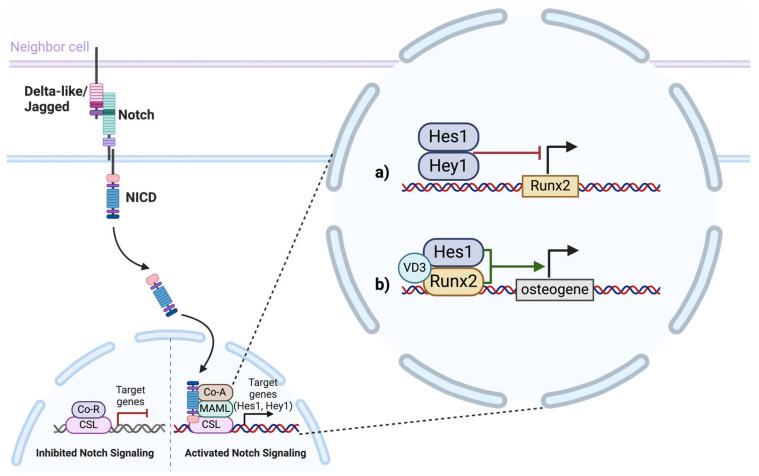
Two possible options for the interaction of Notch signaling pathway targets (Hes1, Hey1) with Runx2. (**a**) Hrt transcription factors (Hes1, Hey1) suppress the transcriptional activity of *Runx2*. (**b**) Runx2 and Hes1 physically interact and enhance basal and 1,25-(OH)_2_-vitamin D3-induced (VD3) transcription of a gene associated with osteodifferentiation. “Created in BioRender. Malashicheva, A. (2024) https://BioRender.com/g44l299”.

Pathological calcification of the aortic valve is a common cause of heart disease among adults. Its incidence increases with age, and this is often associated with a bicuspid aortic valve present in 1–2% of the population [93]. Despite this, the exact mechanisms of aortic valve calcification, as well as the origin of the development of a bicuspid rather than tricuspid aortic valve, are poorly understood. It has been shown that autosomal dominant mutations in *Notch1* cause a spectrum of abnormalities in the development of the aortic valve and its severe calcification [94]. From the data obtained during the study, it was noted that Notch1 suppressed the activity of *Runx2* in the developing aortic valves of mice: Hrt repressors, which are activated by the Notch1 signal, suppressed the transcriptional activity of *Runx2* (Figure 1a). However, with a certain mutation of *Notch1*, an increase in the luciferase activity of *Runx2* was observed, that is, its expression was increased.

Hankenson and colleagues have shown that canonical Notch signaling in bone marrow MSCs can initiate osteoblastogenesis [95]. In addition, the option of direct interaction between Runx2 and RBPJ, the Notch signal modulator, is not excluded. It was noted that the concentration of Notch ligands increased to the stage of cell mineralization on the 10th day of osteogenic differentiation. Suppression of Runx2 expression using siRNA led to a decrease in ALP levels on the 3rd day of osteodifferentiation, despite stimulation of the cells by the Jagged1 and BMP2 proteins, which also inhibited the mineralization of human osteoblasts on the 10th day of differentiation. However, the affinity of RBPJ to the *Runx2* promoter was not detected using the CHIP-seq method in osteodifferentiated human MSCs on day 3, while the ability of RBPJ to bind to the ALP and Osterix promoters was detected. It has also been shown that the administration of DBZ, a gamma secretase inhibitor necessary for blocking Notch activation in vivo, negatively impacts bone regeneration in mice, even with an implant containing BMP2. Therefore, if we are discussing BMP–Notch crosstalk, it is reasonable to investigate the Runx2–Notch interaction, as this study suggests that there may be intermediaries involved in signal transmission.

Studies of differentiation and hypertrophy of mouse chondrocytes and ATDC5 chondrogenic cells have shown that the Notch signaling pathway plays a crucial role in their proliferation and differentiation: Overexpression of NICD, the intracellular domain of Notch, induced the arrest of the G0/G1 phase of the cell cycle and subsequent differentiation of chondrocytes, as determined by flow cytometry [96]. There was also a significant decrease in the expression of the early chondrogenic marker, *SOX-9*, and the expression of regulators and markers of chondrocyte hypertrophy, *Runx2*, *ALP,* and *Mmp13*, was enhanced in NICD-expressing cells. In contrast, treatment with a Notch inhibitor, DAPT, reduced both endogenous and BMP2-induced phosphorylation of SMAD 1/5/8, and knockdown of these SMADs disrupted NICD-mediated differentiation of chondrocytes. Taken together, these results demonstrate that the Notch signaling pathway induces cell cycle arrest, thereby initiating chondrocyte hypertrophy through BMP/SMAD-mediated regulation. In another study analyzing the differentiation of rat chondrocytes treated with the exogenous recombinant Jagged1 protein, the Notch ligand, an increase in the expression of Runx2 was found at both the mRNA and protein levels, while treatment with DAPT led to a decrease in the expression of Runx2 [97].

The role of the transcription factors Hes and Hey, which are Notch targets, has been studied in different in vivo models. Transgenic mice overexpressing *Hes1* exhibited osteopenia (low bone density) due to impaired osteoblast function. Conversely, deletion in *Hes1* in mature osteoblasts led to an increase in the volume of trabecular bone, the number of osteoblasts, and the rate of mineral attachment [98]. The skeletal phenotype of transgenic mice, in which *Hey1* was universally absent or overexpressed, was also analyzed. *Hey1* deficiency led to moderate osteopenia and an increase in the number and activity of osteoclasts cultured ex vivo. Overexpression of *Hey1* led to distinctly progressive osteopenia and inhibition of osteoblast functions ex vivo. In both Hey1-deficient and overexpressed mice, males were less affected than females [99].

*Hes1* is known to be co-expressed with *Runx2* in osteoblastic cells, and Runx2 and Hes1 physically interact [100,101]. In osteoblastic cells, Hes1 was found to enhance basal and 1,25-(OH)_2_-vitamin D3-induced transcription of *OPN* (Figure 1b). This enhancement was suppressed by AML-1/ETO, a Runx2 inhibitor. Immunoprecipitation assays have shown that Hes-1 and Runx2 interact and that 1,25-(OH)_2_- vitamin D3 may enhance this interaction. Together, these data determine the intersection of three pathways—osteogenic differentiation under the control of the Runx2, 1,25-(OH)_2_-D3 pathway and Notch signaling, and, consequently, their important role in the processes of osteogenic differentiation and bone remodeling [102].

In vitro, a dose-dependent mechanism of action of the Notch signaling pathway was discovered in stimulating osteogenic differentiation of MSCs of adipose tissue [103]. Osteogenic differentiation was induced in the presence of one of the components of the Notch signaling pathway—NICD, Jag1, Dll1, and Dll4, after dosing with lentiviral transduction. It was demonstrated that osteogenic differentiation was enhanced with NICD and Jag1 transduction in a dose-dependent manner; however, a high dose of both NICD and Jag1 reduced the effectiveness of osteogenic differentiation. NICD dose-dependently increased the activity of CSL luciferase reporter, but a high dose of NICD caused a decrease in reporter activity. A high dose of both Notch components, NICD and Jag1, induced apoptosis. In co-culture experiments, where only half of the cells were transduced with either NICD or Jag1, osteogenic differentiation increased dose-dependently only with NICD, while cells transduced with Jag1 differentiated almost equally regardless of dose.

Analysis of the luciferase activity of the *Runx2* and *SPP1* promoters in interstitial valve cells and adipose tissue MSCs showed a subtle dose dependence in response to Notch induction and the initiation of osteogenic differentiation, which suggests a direct relationship between the level of Notch activation, the expression of these genes, and the corresponding osteogenic induction [104].

All these data may explain the existing contradictions about the role of the Notch signaling pathway in osteogenic differentiation, but, since the dependence of these processes on the dose of the components of the Notch signaling pathway is still being studied, the mechanisms remain unclear.

## 4. The Tissue Specificity of Runx2: Is Runx2 Truly Only a Pro-Osteogenic Factor as It Has Been Suggested to Be?

**Figure 2:**
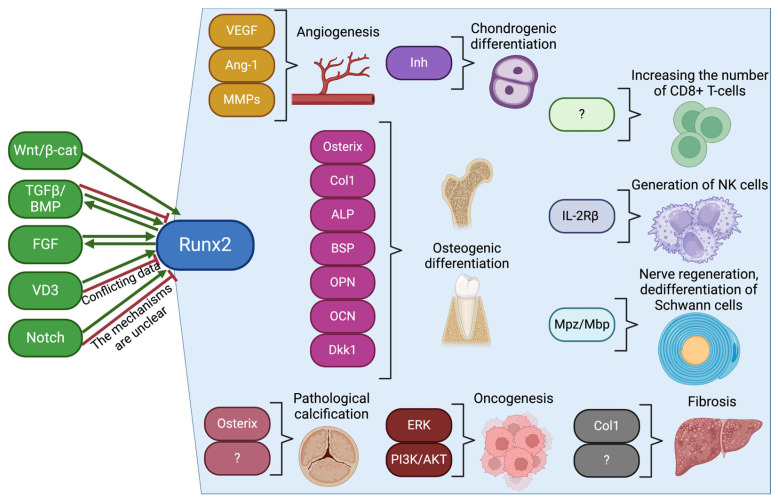
Tissue-specific effect of the Runx2 transcription factor and its associations with various signaling pathways and target genes. “Created in BioRender. Malashicheva, A. (2024) https://BioRender.com/t84w530”.

It is noteworthy that the functional role of Runx2 differs in various cells and tissues, implying that the mechanisms by which Runx2 exerts its functions also differ (Figure 2).

### 4.1. The Effect of Runx2 on Dental Development

Runx2 regulates the mineralization of cells not only in bones, but also in the teeth. FGF4-dependent activation of Runx2 in the epithelium of the tooth germ leads to the launch of FGF3 in the mesenchymal tissue in mice [105,106]. Although Dkk1 expression in osteogenic precursor cells of condensed mesenchyme on day 12 of embryonic tooth development is Runx2-dependent, Runx2 is not required for BMP4-induced expression of Dkk1 in the mandible on day 11. The data demonstrate that FGF induces all genes involved in this process, including Dkk1 and Dusp6, which explains the absence of FGF4 in Runx2 mutants. However, Dkk1 expression depends on Runx2, which indicates that Runx2 may play a role in FGF signal transmission in the dental tissue of mouse embryos [107].

### 4.2. The Effect of Runx2 on Peripheral Nerve Regeneration

Although Runx2 is best known for its role in bone formation, it has other uses as well. For example, single-cell RNA sequencing has shown that Runx2 has a positive effect on Schwann cells, which are associated with peripheral nerves in mice [108]. It was also confirmed that, after damage to the distal nerve in rats, overexpression of Runx2 was observed in Schwann cells in contact with the nerve.

Additionally, it was found that the regulation of Runx2 in mouse Schwann cells is carried out by signaling pathways such as TGF-β1 and FGF5, which interact with Runx2 in other tissues of the body [109]. Activation of these signaling molecules was detected in the regenerating site of a damaged nerve and was associated with the initiation of regenerative processes within cells and tissues.

In response to FGF5 activation, Schwann cells produced Runx2 protein at a higher rate; however, TGF-β1 suppressed Runx2 production. This was confirmed by data showing inhibition of *Runx2* transcription in response to TGF-β1 activation in rats in vivo [110].

In addition to triggering regeneration in case of damage to the peripheral nerves, Runx2 is also required for the dedifferentiation and reprogramming of Schwann cells into mesenchymal progenitors. This process is regulated by the c-jun protein [109]. In human osteoblasts, c-jun also interacts with Runx2 only as an AP1 dimer, together with c-Fos as a transcription coactivator [41]. Moreover, Runx2, which is not expressed during nerve development, is involved in repeated myelination after injury, enhancing the expression of genes encoding myelin proteins in rats (Mpz, Mbp) [109].

### 4.3. The Effect of Runx2 on Angiogenesis

Another role of Runx2 has been found in human endothelial cells, and it is associated with their proliferation and cell cycle control. In the G2 and M phases, the level of endogenous Runx2 protein and DNA-binding activity specifically decrease, which suppresses cell proliferation and delays the cell cycle in humans [111]. The target genes of Runx2—vascular endothelial growth factor (*VEGF*), angiopoietin-1 (*Ang-1*), *BSP*, *OPN,* and matrix metalloproteinases (*MMPs*)—promote the differentiation, migration, and invasion of endothelial cells during angiogenesis [112], which is crucial for tumor growth and metastasis. Runx2 is a potential therapeutic target for bone metastasis due to its role in regulating the behavior of tumor cells and stroma in humans [113]. Inhibition of Runx2 can directly affect tumor cells or disrupt the tumor microenvironment [114].

### 4.4. The Effect of Runx2 on Oncogenesis

Recently, there has been increasing evidence that Runx2 has oncogenic properties in various types of cancer. Data indicate that Runx2 may play a critical role in the development of malignant tumors, metastasis, angiogenesis, proliferation, and resistance to antitumor drugs [115]. It is known that metastasis and low sensitivity to chemotherapy in human osteosarcoma correlate with overexpression of Runx2 [116]. In melanoma, Runx2 enhances tumor invasion and metastasis via the PI3K/AKT signaling pathway [117]. Runx2 was highly expressed in cells and tissues of human esophageal carcinoma, and it also activated the PI3K/AKT and ERK signaling pathways, contributing to tumor growth and progression [118]. Dysregulation of *RUNX* genes, including *Runx2*, can lead to genome instability and disruption of DNA repair mechanisms in both leukemia and single tumors in human [119]. Using breast cancer cells, it has been shown that Runx2 is associated with tumor progression, its spread, and poor prognosis of the disease. It regulates the properties of cancer stem cells and promotes oncogenesis through the process of epithelial-to-mesenchymal transition [120]. In general, all of these different sources note the overexpression and oncogenicity of the Runx2 factor in various types of cancers.

### 4.5. The Effect of Runx2 on the Development of Fibrosis

In the liver, Runx2 is an activator for stellate cells in human fibrosis, and Runx2 knockdown in mice leads to a decrease in fibrotic processes in the organ [121]. In addition, it is known that Runx2 contributes to the development of vascular fibrosis in diabetes mellitus in mice in vivo [122]. A study on the tissues of the aortic valve of the heart of mice demonstrated that when animals consumed food with a high content of cholesterol and fats, the expression of Runx2 increased, accompanied by an increase in the production of ECM, in particular Col1, while calcification did not occur [123].

Ultimately, Runx2 may be one of the options for therapeutic targets in the treatment of fibrosis.

### 4.6. The Effect of Runx2 on Immune Cells

Increased expression of Runx2 at the β-selection stage in the developing mouse thymus led to an increase in the number of immature CD8+ T cells [124]. And in human natural killers (NK cells), it was found that the transcription factor Runx2 positively regulated their generation, affecting progenitors and inducing an increase in the expression of β-subunit of the interleukin-2 receptor (IL-2Rβ) [125]. In addition, Runx2 plays a role in the fixation of NK cells in tissues, that is, it leads to an increase in resident NK.

### 4.7. The Effect of Runx2 on the Development of Spinal Tissues

There is also a study that suggests that Runx2 plays a crucial role in the growth and development of intervertebral disc tissues during the postnatal period in mice. It has been established that a signaling molecule known as Ihh (Indian hedgehog) plays a significant role in maintaining the proliferation and differentiation of cartilage cells [126]. Ihh signaling is essential for the formation of spinal structures, particularly the nucleus pulposus and outer fibrillar ring. Runx2 regulates Ihh expression, and it is a critical factor in determining the fate and differentiation of progenitor cells into pre-chondrocytes within the central pulposus tissue of mice [127].

### 4.8. The Relationship Between Pathological Calcification and the Runx2 Transcription Factor

If physiological calcification occurs during bone formation to strengthen tissues, pathological calcification does not have a clear function and is thought to be associated with tissue abnormalities or with impaired metabolic levels of serum calcium and phosphate ions [128,129,130,131].

Previously, it was believed that vascular calcification was a passive process caused by the deposition of minerals from the circulation, especially in patients with mineral imbalance. However, pathological calcifications are currently associated with osteo/chondrodifferentiation of human cells in the affected areas [132]. Osteo/chondrocytic transformation of vascular smooth muscle cells and stem cells both in vitro and in vivo is accompanied by increased regulation of the transcription factors Runx2, Osterix, MSX2, and SOX-9, which are centrally involved in osteoblast differentiation and chondrocyte maturation. It is also known that in case of thoracic aortic aneurysm in endothelial cells, in response to mechanical stress, the transcription level of the proosteogenic factor BMP2 increases, which stimulates the expression of osteogenes in vascular smooth muscle cells. An increase in BMP2 levels in an aortic aneurysm may indicate that signaling pathways responsible for pathological vascular calcification contribute to the development of an aneurysm in humans [133,134].

In addition, there are experimental models of a bioengineered valve for rats through which *Runx2* expression is suppressed in the endothelium of heart valves, which leads to inhibition of the calcification of heart valve tissue [135].

In general, various discussions have been going on about vascular calcification for a long time. Recently, the mechanisms of calcification in different human arteries have been analyzed, and it has been revealed that the mechanisms of calcification in the tibial and coronary arteries may have common risk factors, as well as specific molecular differences [136]. However, Runx2 levels were significantly increased in the tibial artery and in the human aorta, but not in the coronary artery, indicating that the mechanisms of pathological ossification differ in different vascular tissues. Aortic stenosis associated with pathological calcification of the aortic valve was studied on cells derived from patients with bicuspid or tricuspid valves [137]. It was revealed that the calcification mechanisms are different in these patients, and the Notch signaling pathway plays an important role in these processes.

## 5. Conclusions

Runx2 undoubtedly performs various functions in different cells and tissues of the body. Despite the fact that Runx2 has traditionally been known as the main regulator of normal osteogenic cell differentiation, the answer to the question of why this happens lies in the study of the mechanisms of interaction between Runx2 and its protein and gene targets. It is important to understand at what level and how Runx2 regulates these processes: at the level of transcription, translation, or through numerous post-translational modifications. Additionally, there are numerous unanswered questions regarding the processes in which Runx2 plays a direct role. Although Runx2 is generally recognized as a factor primarily responsible for basal bone formation, it also has the potential to regulate the cell cycle and, in some instances, lead to pathological calcifications and oncogenic and fibrotic alterations in various cells and tissues. An intricate interplay between Runx2 and various signaling pathways underscores the significance of this factor for the proper functioning of the organism. It is evident that the interaction between Runx2 and the Notch signaling pathway occurs at multiple levels. However, the exact mechanism of how specific processes are regulated at the molecular level remains uncertain and requires further investigation. Runx2 represents a significant area of research interest and has the potential to become a valuable therapeutic target for many pathologies.
